# International youth mental health case study of peer researchers’ experiences

**DOI:** 10.1186/s40900-023-00443-4

**Published:** 2023-05-15

**Authors:** Inga Spuerck, Milos Stankovic, Syeda Zeenat Fatima, Elmas Yilmas, Nicholas Morgan, Jenna Jacob, Julian Edbrooke-Childs, Panos Vostanis

**Affiliations:** 1 Euro Youth Mental Health, London, UK; 2 Hussaini Foundation, Karachi, Pakistan; 3 KTO Karatay University, Karatay, Turkey; 4 Anna Freud Centre, London, UK; 5 University of Leicester, Leicester, UK; 6 University of Johannesburg, Johannesburg, South Africa; 7 University College London, London, UK

**Keywords:** Participatory research, Peer researcher, Youth mental health, Lived experience, Majority world countries

## Abstract

**Background:**

The involvement of young people as peer researchers, especially with lived experience, is increasingly considered important in youth mental health research. Yet, there is variation in the understanding of the role, and limited evidence on its implementation across different research systems. This case study focusses on the barriers and enablers of implementing peer researcher roles within and across majority world countries contexts.

**Methods:**

Based on an international youth mental health project involving different levels of peer researchers and participants from eight countries, peer researchers and a co-ordinating career researcher reflect on lessons regarding enabling and challenging factors. These reflections are captured and integrated by a systematic insight analysis process.

**Results:**

Building on existing international networks, it was feasible to actively involve peer researchers with lived experience in a multi-country mental health study, who in turn recruited and engaged young participants. Identified challenges include the terminology and definition of the role, cultural differences in mental health concepts, and consistency across countries and sites.

**Discussion:**

Peer researchers’ role could be strengthened and mainstreamed in the future through ongoing international networks, training, sufficient planning, and active influence throughout the research process.

*Trial registration*: Not applicable.

**Supplementary Information:**

The online version contains supplementary material available at 10.1186/s40900-023-00443-4.

## Abstract (summary)

The involvement of young people with lived experience as collaborators (peer researchers) in mental health research has become more prominent in recent years. Yet, there is variation in the understanding of this role and how to involve young people. There is also limited evidence on how this role can be applied across different research systems, especially in majority world countries. We share our experience from a youth mental health project involving peer researchers in eight countries. Peer researchers and the co-ordinating career researcher reflected on lessons regarding enabling and challenging factors. Their reflections highlighted that, overall, it is feasible to successfully involve young people with lived experience in international mental health research. Several challenges were also identified, including terminology, definition of roles, cultural differences in understanding mental health, and consistency across participating countries and sites. According to peer researchers’ reflections, these challenges can be overcome through the establishment of ongoing international networks, training and support, sufficient planning and peer researchers getting involved at all the stages of the research. Lessons from this case study can be of interest to the international research community in strengthening the involvement of young people in mental health research.

**Supplementary Information:**

The online version contains supplementary material available at 10.1186/s40900-023-00443-4.

## Background

In the last few decades, different approaches of participatory research have been developed and reported [1]. Common to variably defined approaches is that people who are the focus of the research are involved as collaborators in the research process, instead of them merely being participants [1, 2]. While some of these approaches such as community based participatory research (CBPR) or participatory action research (PAR) aim to produce solutions arising from the research findings, co-production predominantly focuses on the generation of new knowledge [3]. A widely used term for co-production is ‘peer-research’ or ‘co-research’. However, the definitions of peer researchers and the levels of their involvement are inconsistent across the literature. Participation broadly consists of information, consultation, involvement, collaboration and empowerment [1].

The umbrella of co-production approaches includes concepts such as ‘patient and public involvement’ (PPI) in health research, ‘service user research’ across health and social care, and ‘peer research’. For example, patient and public involvement refers to consultation and collaboration with experts by experience (research carried out with or by members of the public rather than to, about or for them) [4], whilst peer researchers are experts by lived experience who conduct pre-defined components of the research. Peer researcher activities may include designing, delivering, analysing, interpreting, or disseminating aspects of the data they have generated; and identifying actions [5]. Nevertheless, these roles vary in the literature, and related terms are used inter-changeably. Lack of consensus possibly applies even more to the role of young people as peer researchers. This is both because of the relatively recent body of studies in this field, as well as because of developmental and ethical challenges [6, 7].

### Involving young people as peer researchers^1^

The rights of young people to be involved in decisions affecting their lives is widely acknowledged, as highlighted by the United Nations Convention on the Rights of the Child [8]. To ensure that research findings are responsive to the needs of young people, there has been increasing interest in their meaningful involvement in research, i.e., their active input rather than tokenistic presence without their views being taken into consideration [9]. A growing body of evidence indicates several positive effects of involving young people as peer researchers on the youth-centredness and quality of the research process at all stages.

Because of their peer networks, young people can help with recruitment, especially of hard-to-engage groups, therefore, extend the pool of potential participants [7, 10, 11]. Involvement in data collection, e.g., by interviewing other young people, can reduce power imbalances between researcher and participant, and as a result, the bias of collected information. Peer researchers share similar experiences and language, which may lead to participants feeling more comfortable in sharing their views. Analysis and interpretation of data in collaboration with young people can add a new perspective, thus enhance our understanding of the subject; whilst dissemination of findings to their peers can enhance the uptake of solutions, hence the impact of the research [11, 12]. Overall, the active involvement of young people as peer researchers can benefit both the young people by increasing their self-confidence and them feeling that their views matter, *and* the career researchers by enriching their insight into young people’s needs and perspectives [13].

Despite these benefits, the question of how to meaningfully involve young people in research remains a matter of debate, with various emerging peer researcher models [9]. Most authors agree that youth involvement in research needs careful consideration according to each research context [7, 14, 15]. This process requires flexibility and adaptation by the career researchers regarding the peer researcher role, required training and support, timescale, and budget [7, 14]. To prevent any arising risks of harmful participation, a range of ethical issues should be considered and managed appropriately. These include the safeguarding of vulnerable young people, ensuring confidentiality, and incorporating professional payment rather than potentially coercive rewards [10].

Researchers need to have the necessary skills to build good rapport with young people and to respond to their needs in relation to, e.g., life changes, puberty, or school commitments [15, 16]. McLaughin [11] recommends that the research process should allow for young people to leave and re-join the project if faced with personal challenges. It is thus essential that research teams sustain ongoing dialogue among everyone involved, whilst offering training and other support to peer researchers [14]. Brady and colleagues [17] argue that researchers should give young people an informed choice to decide whether and how they want to be involved, especially when conducting research on sensitive topics such as abuse, interpersonal violence, or exploitation, which may cause distress to both young interviewers and interviewees [15]. Mental health is another sensitive topic, because of the attached stigma, and the potential of triggering adverse experiences and emotions. Therefore, it is important to consider the additional requirements when involving young people as peer researchers in mental health related research.

### Peer researchers in mental health studies

Evidence on the involvement of young people as peer researchers in mental health research mainly originates from high-income or minority world countries [9, 14, 18, 19]. Important lessons also arise from the large body of literature on youth engagement in mental health research [20]. Overall, evidence indicates that the involvement of young people with lived experience of mental health difficulties in research is feasible, provides valuable insight [13, 18], enhances empathy [21] and contributes to stigma reduction [22]; also, that young people, as well as career researchers, are motivated to engage with each other [18, 23]. The Recovery Colleges approach of involving students with lived experience was found to lead to interpersonal changes and use of opportunities [24]. Delman [25] stated that meaningful youth involvement should be underpinned by the key principles of personal commitment to leadership, inclusion, respect, clear communication, project flexibility, additional resources, supportive infrastructure and training.

These promising findings also highlight the importance of providing adequate support to peer researchers in dealing with ongoing mental health and age-relevant issues [26, 27]. If young people are feeling less confident because of mental health difficulties, it may take more time and encouragement from career researchers to involve them positively in a research project [23]. This means that career researchers themselves require training, irrespective of seniority and previous experience, as a youth participatory process would require new skills [26]. To this effect, Faithfull and colleagues [18] found that the degree of confidence and competence career researchers felt about engaging with young people influenced how they conceptualised youth participation.

Several challenges have thus been identified in relation to mental health research. At the planning stage, it is usually suggested to over-recruit peer researchers, as the risk of drop-out is higher due to the potential of deteriorating mental health difficulties, changes in life circumstances, and education or employment opportunities [14]. However, the recruitment of peer researchers with lived experience of mental health difficulties can be more challenging than in non-mental health related research, as young people might fear that their involvement could face stigmatising attitudes from the research team or stakeholders such as professionals; and lead to the deterioration of existing or the recurrence of previous mental health difficulties [23]. As similar fears were found to arise from young people previously experiencing discrimination and marginalisation, it is essential to allow for building trust with the other researchers over a longer period of time [28]. Consequently, a peer researcher with lived experience can feel being both ‘insider’ and ‘outsider’ in the research process [29].

### Peer researchers in majority world mental health research

Although the limited evidence is based on minority world sociocultural contexts, participatory methods to involve young people in mental health research have been implemented in majority world countries (MWC). Approaches used in MWC were predominantly CBPR or PAR, rather than being based on the peer researcher model. These studies made a contribution to the involvement of peer researchers in international mental health research by understanding cultural norms, thus tapping into expert knowledge and maximising available resources, and empowering participants [30]. Most studies involved adults with mental health or disabilities [e.g., 31, 32.

In relation to youth mental health, Afifi et al. [33] followed the CBPR approach in a mental health study in Beirut, the multi-cultural capital of Lebanon, and established that lack of understanding of gender roles or patriarchal structures by the researchers hindered young people from openly sharing their opinions. Stacciarini et al. [34] also used the CBPR approach to explore the conceptualisation of mental health by minority populations. Consistent with other studies, concepts varied in different parts of the world, from ‘wellness’ to ‘mental illness’. Consequently, the authors suggested that the design of both culturally appropriate mental health research and interventions should be informed by feedback from community members, whilst also keeping the cross-cultural comparability of studies in mind. As underserved populations in MWC were often involved as just the ‘objects’ of academic research, usually with little benefit to their communities, there might be additional barriers in recruiting and truly involving young people as peer researchers in the growing body of international youth mental health research [33].

In summary, even though participatory methods are increasingly used in mental health research, to our knowledge there are no studies reporting on the participation of young people as peer researchers in international mental health research involving multiple MWC. Understanding and addressing the specific requirements of planning, conducting, and disseminating research with peer researchers in MWC is important in establishing generalisable evidence, thus improving youth mental health in resource-constrained settings across the world. This research gap informed the rationale for this case study, in the context of an international youth mental health participatory project.

## Aim of the case study

In this case study, we reflect on the experiences of young people who were involved as peer researchers in an international mental health project, and further discuss best practices of youth involvement in sensitive research. We particularly focus on barriers and enablers of implementing peer researcher roles within and across MWC contexts. The wider objective is to clarify, strengthen and mainstream the role of peer researchers. To this effect, lessons from this case study would be of interest to peer and career researchers, governance bodies, policy makers and funders across the research community, especially in an international context.

### Research context of the case study

We summarise the context of the wider research in which peer researchers were involved, before describing the methodology and procedure of this case study. Its objective was to establish the lived experience of young people on the active ingredients (or mechanisms) of common mental health difficulties, depression and anxiety. Details on the project can be found in [35]. We selected MWC that were broadly representative of the socioeconomic spectrum [36]. These consisted of India, Pakistan, Turkey, Kenya, South Africa and Brazil. We also opted to include youth experiences and perspectives from two minority world countries, Portugal and the UK, in order to explore both commonalities and context-specific issues across different systems. Within each country, a non-governmental organisation (NGO—in six MWC) or academic institution (Portugal) or peer-led lived experience charity (UK) acted as local project lead. Each host agency, through their local networks, invited young people aged 14–24 years who had experienced depression and/or anxiety, to participate in focus groups.

Two youth focus groups were facilitated in each country, with an average 6–8 and total 121 local youth participants. Research ethics approval was obtained by the University of Leicester Psychology Research Ethics Committee in the UK. Youth aged 16–24 provided written informed consent. Parents or carers of younger participants aged 14–15 years gave written informed consent, following which young people provided verbal assent. Each focus group was facilitated by a senior member of the host agency, with co-facilitation by a peer researcher from the same organisation. A member of the central research team observed remotely the focus group discussions.

### Role of peer researchers in the overall international research project

Throughout the project, peer researchers were actively involved, to ensure that the research activities and outputs were in line with young people’s understanding and feedback. One peer researcher from each country/site was involved, although their local and central contributions varied. Each local peer research group was co-ordinated by two lead peer and one career researcher (also see below). In particular, local peer researchers:

1. Engaged young people in each partner country and interpreted their feedback, as a result of their cultural expertise.
2. Ensured that communications and materials were tailored to young people and reflected country-specific considerations.
3. Avoided losing sight of the young person’s perspective when interpreting the data.

Engagement practices were informed by PPI guidelines [4]. These objectives were achieved through participation in country-specific research team meetings, co-facilitation of local focus groups, and attendance at three multi-country advisory youth groups. The two lead (central) peer researchers were involved in parts of the research proposal and design. The local (country-specific) peer researchers were involved on confirmation of grant approval, because of the tight schedule between grant application, approval, onset and completion. They had though opportunities to influence both the local and wider context of the study.

## Methodology of the case study

Although we did not follow a research methodology on peer researchers’ roles in parallel to the main project, we adopted a systematic insight analysis process of capturing and integrating young peer researcher and co-ordinating career researcher perspectives in relation to the aim of this case study. Insight analysis is an adopted methodology where evidence is reviewed and interpreted, with inferences derived [37]. This process included: a group directed discussion between three peer researchers from Turkey, Pakistan and Brazil; a directed discussion between the two lead peer researchers; and inclusion of the perspective of the co-ordinating career researcher. All participants considered their understanding of their role, experiences in different aspects of the project, cultural and other contextual issues, links with the central research team, and recommendations for future research. For the purpose of this paper, we asked the researchers who were involved in these discussions to summarise them. Their perspectives and experiences are synthesised as narrative reflections in three respective sections below and are subsequently integrated in an overarching discussion (see Additional file 1: Appendix 1 for topic guide). We followed established guidelines in reporting the involvement of young people with lived experiences (see Table 1) [38].

**Table 1:** GRIPP2 reporting checklist (short form) [38]

Section and topic	Item	Reported on lines no
Aim	To capture enablers and barriers in the involvement of peer researchers in international youth mental health research, especially in MWC	207–215
Methods	Two central and three local peer researchers (from Brazil, Pakistan and Turkey) with lived experiences considered their roles in an international youth mental health project. Their views were integrated through insight analysis	216–279
Results	Enablers included engagement of young participants across different MWC (recruitment, retention and participation), and active input to data collection, analysis and dissemination. Barriers included lack of clarity in peer researcher role and name, cross-cultural conceptual mental health differences, and challenges in maintaining consistency across countries/sites	284–385
Discussion and conclusions	Some findings were universal to the peer researcher role, whilst others were context-specific in relation to youth, mental health and MWC. Overall, peer researchers were positively received across all participating MWC, and their lived experience was essential in relating to young participants and enriching the whole research process. Peer researchers were thus able to bridge ‘insider’ vs ‘outsider’ challenges. Existing partnerships with MWC facilitated peer researchers’ involvement, although these networks were not specific to youth with lived experiences. Not enough time and clarity was built in the research design to define peer researcher roles and ensure their consistency	387–547
Reflections/critical perspective	It is feasible to successfully involve young people with mental health lived experience in international research, particularly if connections through networks and partnerships are already in place. Their role should be clearly defined at planning stage, with built in training, support and costings. Cross-cultural research should additionally reconcile conceptual mental health variation and ensure role consistency. This would be facilitated by the establishment of ongoing global youth peer researcher networks	548–571

### Reflections of local (country-specific) peer researchers

We experienced several benefits and challenges whilst participating in this project. Our roles varied and involved organising and co-facilitating focus groups, helping with transcripts, sending materials, feedback, and opinions to the central research team, and writing a blog. Most importantly, we enabled young participants to express their opinions freely. Working with peers across the world and sharing learning among different countries throughout the research, was a unique experience. Our impression was that young participants felt more comfortable in working with a peer, rather than a career researcher, because of the generation gap: *“It is important to have somebody of your age or little older, because they can empathise with you and understand you more than somebody who is of an older age. If the old researcher has been polishing his skills or is using different techniques, then it’s fine”.* As focus groups should be both formal and comfortable, co-facilitation between a peer and career researcher appeared to achieve this balance.

Other benefits included the acquisition of research skills such as learning how to listen actively and develop critical thinking, dealing with ethics issues, co-facilitating focus groups, interpreting data, and giving feedback. Having access to the central research team and regular discussion forums were helpful in understanding and adapting our role during different stages of the project. Both the structure of research meetings and the ongoing communication with all researchers were important in this.

Taking part in such complex research also brought challenges, particularly for those of us who had not had any previous research experience, not least as peer researchers. The terms ‘peer advisor’ and ‘peer researcher’ are not easy to understand and translate in national languages, so they should be avoided. The best alternative is ‘young leader’. Other terms considered in our discussion were: young advisor, young researcher, young research advisor, youth facilitator, and research organiser.

Despite our similar age, engaging other young people was not always easy, as there were sociocultural barriers, even within the same society. Choosing research topics that are meaningful to young people and communicating their relevance, would increase recruitment and retention. Language constraints in multi-country meetings could improve with the use of visual tools. The facilitation of focus groups was difficult at times, trying to keep young people engaged whilst encouraging them to open up on sensitive mental health experiences, handling different opinions, and moderating between vocal and quiet participants. As allocated time was insufficient, a larger number of focus groups would have allowed the involvement of more diverse young participants. These multiple tasks require training, supervision and ongoing support; for example, in organising demo sessions of facilitating focus groups. These should start at the planning stage, be built in the project, and allow for time and resources.

Peer researchers have an advantageous role in disseminating findings by using creative, therefore engaging, approaches. By sharing findings with professionals and institutions, they can help break previous barriers in communication. Platforms could include online meetings, posters, radio interviews, study circles or multipliers, and other art-based formats. Cross-cultural presentations are particularly important in learning from each other, as well as highlighting similarities. Crucially, peer researchers can lead and/or take active part in implementing findings through psychoeducation and self-help groups.

### Reflections of lead (central) peer researchers

Overall, the involvement as lead peer researchers in this research project was a positive and enriching experience. From our perspective, we believe that youth involvement, does not only have a benefit for the research results (being more related to young people's views), but also benefits young people’s personal development, e.g. feeling empowered.

We did not experience that the sensitive mental health topic was a barrier in this research project, but we did see that the concepts of mental health and mental health diagnosis are understood differently in different countries. Therefore, we needed the help of the local peer and career researchers to find appropriate wording that is understood in the specific country. In general, language was a challenge at some parts of the project. For example, some local focus groups were conducted in the predominant national language, so we were not able to follow along what was talked about and be supportive.

When we joined the central research team, most of the planning work had to be completed quickly, because of the strict time schedule of the project. Even though we were able to be part of the planning process, more time would have been helpful for us. Therefore, in the beginning it was challenging for us to understand our role as lead peer researchers and the roles of everyone else involved: central researcher, central co-ordination researcher, local career researchers, and local peer researchers. While having regular meetings with the co-ordinating career researcher, sometimes it would have been more helpful to have meetings with a career researcher being involved in the actual tasks we were involved in, such as data collection. But over time and through regular meetings with the whole central research team, our tasks and responsibilities became clearer. We both already had some previous research experiences, which also helped us in understanding the structure of the project.

Therefore, we suggest that, if peer researchers have never been involved in research projects, they should first attend basic training in research skills. Additionally, by involving peer researchers in the phase of research preparation and planning, the problem of not understanding their role could be prevented or at least confusion could be reduced. In general, we think that, for fulfilling the role as a peer researcher, it is helpful to have a space to speak about difficulties that come up during the project, discuss the content but also the way the project is managed, and formulate proposals on how to improve future research. To involve more young people as peer researchers in mental health research, it is important to inform young people about research, how research can impact on practice but also on their everyday life, and what difference their involvement can make.

### Reflections of co-ordinating career researcher

The biggest issue that stuck out for me was related to both the varied differences and similarities among our peer researchers. Cultural experiences and how they impacted on their own and their peers’ insights across the research were incredibly useful. Also, working alongside our lead peer researchers ensured an element of consistency and youth perspective throughout our research narrative.

As someone who has worked in the field of youth participation in mental health, there was a range of understanding to the terminology and concepts of participation in research and ‘peer researchers’ at the start, which needed to be discussed and agreed upon. In future projects like this, thorough and effective training is needed to ensure that peer researchers are working from the same page, and that they feel empowered and comfortable sharing their insights and opinions.

Youth participation in general still needs a lot of awareness-raising and training for researchers and stakeholders. I do believe that the future of research rests in peer researchers, and the direct participation of young people in research design and initiation. Those who live certain experience know the topics and areas that need further research, as well as the methods and means of data collection, using current and up-to-date tools and resources.

## Discussion

The aim of this case study was to reflect on the experiences of young people who were involved as peer researchers in an international mental health project, and to further consider best practices of youth involvement in mental health research. It contributes to the field by demonstrating, through triangulation of sources, how young people with lived experience can co-produce solutions with mental health professionals and researchers. These principles can be applied in any local context, as well as internationally, especially in resource-constrained MWC contexts with limited research experience. The reflections of these key actors highlighted that, overall, it is feasible to successfully ‘connect’ young people with lived experience in international mental health research, whilst also identifying certain ‘disconnections’ that need further attention by researchers around the world.

### Role definition

Participants reported that the definition of the peer researcher role in the project was not clear at the beginning, hence it led to confusion. While there is acknowledgement in the participatory research literature that terms and concepts vary [1], these reflections reveal two major points that appeared to contribute to lack of consistency in our project. Local peer researchers pointed out that the term ‘peer researcher’ was difficult to translate into national languages. As concepts can be understood differently in different languages, a good translation does not only include literal meaning but also requires contextual interpretation [39, 40]. Collins and colleagues [41] acknowledged this disconnection, which goes beyond semantics, in that research and thematic terminology may often prove disengaging for some young people. Even though local peer researchers are experts of their own culture, therefore have the contextual background to interpret terms correctly, they may be new to research. It could thus become even more difficult for them to translate research-related terms into their language. Therefore, it is important to develop consensus on the term and underpinning role among all senior and peer researchers from the outset.

### Role components

Lead peer researchers reflected that the use of several titles of career and peer researchers by the central and local research teams made it more difficult to understand differences in responsibilities and tasks. This may have been compounded by the fact (or resulted in) that the actual tasks varied between peer researchers. For example, some peer researchers were predominantly involved in organising and leading focus groups, while others helped mainly with preparing materials or interpreting data. However, giving peer researchers a choice in how they want to be involved—which can then lead to varying roles—has been preferred over having pre-defined roles by some researchers [17].

Another aspect that makes it more challenging to define and understand the role is the dilemma of them being caught between being viewed as ‘insider’ by peers and at the same time as ‘outsider’ by career researchers. The international context of this study added a further insider–outsider challenge, as local peer researchers can be seen as insiders of their cultural context but have an outsider’s perspective in relation to peer researchers from other countries. Kanuha [42] reconciles this contradiction as, in studying one’s own identity group (in our case young people), peer researchers need to maintain connection to their identity group, while at the same time distance themselves. This can be a difficult experience, at personal and professional level. Kara [43] evaluated the value of mental health service user involvement, and argued that such conflicting roles may produce resistance, but can also enrich individual and collective experience.

Several lessons from this project appeared universal to the peer researcher role, rather than context- or culture-specific. The overarching objective of involving peer researchers is to make findings more responsive to the needs of young people, thus impact on societal attitudes, practice, service development and policy. This requires their active influence throughout the research process. The involvement of lead and local peer researchers ensured that the youth perspective was represented consistently at all stages of the project. Several benefits were highlighted in relation to the organisation and delivery of the research. A unique contribution was the mediation between young participants and researchers, as well as the ‘translation’ and reframing of their perspectives. Recruitment, engagement and retention of young participants was particularly facilitated and enhanced by peer researcher input. Local peer researchers described that they could improve the quality of collected data, as young participants felt more comfortable sharing their mental health experiences with someone of their age. Empathy between the two young groups appeared to relate both to their mental health experience and life stage. For similar reasons, peer researchers indicated their advantageous role in the dissemination of results through youth-friendly and creative approaches that communicate positive implications for young people’s mental health.

### Role application in mental health research

Lead peer researchers reflected that there was a culturally dependent understanding of the concept of mental health across participating countries. While different concepts of mental health are also existing within one country, these differences become more prevalent in cross-country research [44]. Mental health concepts could originate from variable levels of mental health awareness, stigma, and access to services [45]. Because of stigma and limited availability of mental health services in some countries, only few young people receive a diagnosis and appropriate support. These issues can make it more difficult for peer researchers to recruit and engage participants with lived experience. For those reasons, we included participants with ‘self-identified’ experience of anxiety and/or depression. This may have reflected a range of mental health needs and experiences of services, especially in MWC.

Peer researchers had limited information on which to engage young participants, without delving into sensitive personal issues or creating unnecessary distress through different understanding of, e.g., the meaning, causes or implications of a condition such as depression. In anticipation, we took into consideration previous evidence that terms like ‘wellness’ or ‘mental health promotion’ are culturally more acceptable, can help open up conversations and reduce stigma in minority and non-western populations [34].

The sensitive concept of mental health did not appear to be a barrier in this study. One reason, as suggested by local peer researchers, could be that a topic needs to be relevant to young people’s lives to motivate them to participate. Our information letters, communication and focus groups guides were designed to explore everyday implications of mental health difficulties and required support. Demonstrating such relevance to young people, who did not necessarily have many opportunities to share their own experiences and suggestions, can enhance their willingness to contribute, in order to help others, through the dissemination of the findings.

### Developing the peer researcher role in an international context

As lead peer researchers reflected, several strategies need to be put in place to enable local peer and career researchers to approach, reassure and meaningfully involve young people in sharing their unique expertise across different cultures. Offering peer researchers different levels of involvement can strengthen their feeling of being heard, therefore raise engagement. Methods should be flexible and adapted to young people’s needs and preferences [46]. Although career researchers should drive, plan and organise such involvement, Faithfull et al. [18] found that their confidence to engage young people depended on their understanding of youth participation. Consequently, awareness-raising and training for career researchers is essential in this process, with significant contribution from peer researchers. When these strategies are put in place, there is evidence of positive impact on both peer and career researchers’ personal development [47].

In future research, it would be helpful to involve local peer researchers at an earlier stage of the project. Peer researchers would then have more time to find into their roles, before starting the main tasks like data collection. This would also enable them to be part of the process of defining and agreeing shared language regarding research terminology. Clear and regular communication between all research actors, especially when multiple countries and/or sites are involved, is paramount in ensuring role coherence and comparable outputs [14]. Training and ongoing supervision of peer researchers, in conjunction with cross-site forums, can help ensure youth recruitment, compliance with ethics standards, and fidelity of collected data, whilst allowing for cross-cultural variation of mental health concepts and support systems.

Even though we were able to engage a total of 12 peer researchers, the reflections of lead peer researchers, local peer researchers and co-ordinating career researchers highlight the importance of a more systematic approach on how to recruit and engage young people in this role, especially in settings where this is less understood and established. Involving peer researchers with lived experience would not have been possible without the collaboration of two international networks. The central (lead) peer researchers and co-ordinating career researcher were leading members of the Euro Youth Mental Health Group network, which generates expertise on different aspects of advocacy, mentorship, education and research. The World Awareness for Children in Trauma network provides access to both peer researchers and young participants in MWC through partnerships with host NGOs and local researchers, who have been involved in youth mental health capacity-building and research [48].

Although hosting organisations and researchers were not initially familiar with the peer researcher role, these networks had already established a working relationship with the central research team, were open to innovation and, crucially, shared a youth-centric philosophy. International (indeed national or local) research networks should involve peer researchers, and constantly monitor their evolving role, training and support needs, and available funding. For the same reason, peer researchers should be central to funding bodies and panels, with meaningful rather than tokenistic input to decision-making. They should have opportunities to disseminate their research in their own right, rather than through career researchers.

### Limitations

These considerations should be interpreted within certain constraints of this case study. Although peer researchers had a central role in the wider project, the evaluation of their role was not included in the design and data collection. This would have been an interesting parallel process. The small and heterogenous sample is not necessarily generalisable to other populations. Similarly, the reported reflections were only made post-hoc. It would be interesting for future research to capture peer researchers’ views and experiences throughout a project by collecting reflective diaries. We only included reflections of some local peer researchers, hence not all cultural perspectives were captured. A self-selection bias may have occurred, as those who participated in the post-hoc insight analysis may have had a more positive experience. Allowing for time and budget early in the research process would have enabled the local peer researchers to influence the research design. If peer researchers are not actively and meaningful involved throughout all stages of a research project, this can create a power imbalance that will negate their influence on the findings and implications of the youth-focused research. Nevertheless, the reported perspectives provided a valuable first insight into the experiences of peer researchers participating in an international project. The complex organisation and completion of this study by involving peer researchers from eight countries highlighted a number of enabling and challenging factors, especially in majority world countries contexts, which can inform the further development of the role.

## Conclusions

To our knowledge, this is the first paper reporting on the experiences of peer researchers in an international youth mental health study. Reflections present an overall promising picture and indicate that it is feasible to successfully involve young people with lived experience in cross-cultural or cross-country research, particularly if connections through networks and partnerships are already in place. Lessons from this project also highlight existing or potential disconnections, and how these can be anticipated and addressed. Strategies could involve a clear structure and orientation, while remaining flexible in enabling young people to influence the peer researcher role. International studies could use standardised concepts of mental health, but also remain aware and sensitively reconcile cultural differences. This is especially important if peer researchers and young participants have lived experience. Peer researchers’ motivation could be enhanced and sustained through a systematic approach to the research, training, supervision and financial support. Allowing for time at the planning stage could help delineate different aspects of the role such as local recruitment of young participants and data collection, as well as co-ordination across countries and sites. Peer researcher input would be more meaningful through co-production with other research stakeholders. While high quality research should strive for methodological fidelity, the involvement of peer researchers from minority and majority world countries with variable research systems and youth participation, requires flexibility and adjustment to cultural, systemic and professional contexts. The parallel development of international young peer researcher networks, ongoing training and infrastructure can contribute to high quality outputs with, crucially, lasting impact for young people in need.

## Supplementary Information

**Additional file 1**. Topic guide of issues explored with peer researchers.

## Data Availability

Not applicable.

## Abbreviations

MWC: Majority world countries
PPI: Patient and public involvement
CBPR: Community based participatory research
PAR: Participatory action research

## Footnotes

1 In this paper we are using the term ‘peer researcher’, as this closely reflects the focus of the role addressed in the paper, whilst acknowledging the variation of terms used in the literature; and the term ‘career researcher’ to refer to researchers across career stages, who are employed by an academic or other research institution.
