# Supplementary material for: International youth mental health case study of peer researchers’ experiences
Source: Res Involv Engagem. 2023 May 15;9:33. doi: 10.1186/s40900-023-00443-4 (PMC10186639; doi:10.1186/s40900-023-00443-4)
Supplement: Supplementary file 1 — Additional file 1. Topic guide of issues explored with peer researchers. [file 40900_2023_443_MOESM1_ESM.docx]

**Appendix 1**

**Issues explored by peer researchers, in relation to their role**

**in a global youth mental health project**

- Could you reflect on your experience from the project? What sticks out most? Found most interesting/useful? Least useful?
- When you look back, were you clear of your role at the beginning of the project? Did it become clearer as the project evolved? How/Why?
- Were there ideas that you were able to bring in? How about your peers?
  Give example. Reasons?
- Were there aspects of the role that were more/less appropriate to your culture/country? Why? How could they be improved in the future?
- How about the research team? What was useful or could have been handled better to help you in your role?
- Had you heard of youth participation (or similar term) in mental health research before? In what way?
- From your recent experience, how could it be developed and broadened in the future? What barriers do you anticipate (e.g., with researchers or other stakeholders in your country), and how could these be overcome?
- How could peer researchers influence a research project from its planning stage to its completion? Examples? Why?

*Prompts:* design, sampling, recruitment, engagement, data collection.

- How could peer researchers make mental health research with children and youth more ethical?
- How about their role in interpreting and understanding the findings?
- How could peer researchers help disseminate the findings? What kinds of sharing knowledge would you recommend? How? Who for?
- How close should peer researchers be in matching the characteristics and lived experiences of youth participants? Pros and cons?
- What specific issues should we consider in relation to *mental health* research? Strengths? Risks?
- What support would peer researchers need to fulfil their role?
  *Prompts*: training, supervision, financial or future career incentives.
- Any other ideas on how young people like yourselves can become more meaningfully involved in mental health research, especially in your country?
